# New Perspective for Non-invasive Brain Stimulation Site Selection in Mild Cognitive Impairment: Based on Meta- and Functional Connectivity Analyses

**DOI:** 10.3389/fnagi.2019.00228

**Published:** 2019-08-27

**Authors:** Jiao Liu, Binlong Zhang, Georgia Wilson, Jian Kong, the Alzheimer’s Disease Neuroimaging Initiative, Michael W. Weiner

**Affiliations:** Author Affiliations: UC San Francisco; University of Southern California; UC San Francisco University of Southern California Mayo Clinic, Rochester Mayo Clinic, Rochester; UC Berkeley; U Pennsylvania; USC; UC Davis; Brigham and Women's Hospital/Harvard Medical School Indiana University Washington University St. Louis University of Pennsylvania; Prevent Alzheimer's Disease 2020 (Chair) Siemens; Alzheimer's Association University of Pittsburgh Washington University St. Louis Cornell University; Albert Einstein College of Medicine of Yeshiva University; AD Drug Discovery Foundation; Acumen Pharmaceuticals; Washington University St. Louis; Northwestern University; National Institute of Mental Health; Brown University; Eli Lilly (Chair); BWH/HMS (Chair); University of Washington (Chair); Mayo Clinic, Rochester (Core PI) University of Southern California; UC San Diego; UC San Diego; UC San Diego; UC San Diego; UC San Diego; UC San Diego; UC San Diego; UC San Diego; UC San Diego; UC Davis (Core PI); UC Davis; UC San Diego; Mayo Clinic, Rochester (Core PI); Mayo Clinic, Rochester; University of London; UCLA School of Medicine; UCSF MRI; UC Davis; Mayo Clinic; Mayo Clinic; Mayo Clinic; Mayo Clinic; Mayo Clinic; Mayo Clinic; Mayo Clinic; UC Berkeley (Core PI); University of Michigan; University of Utah; Banner Alzheimer's Institute; Banner Alzheimer's Institute; University of Pittsburgh; UC Berkeley; Washington University St. Louis; Washington University St. Louis; Washington University St. Louis; Washington University St. Louis; UPenn School of Medicine; UPenn School of Medicine; UPenn School of Medicine; UPenn School of Medicine; UPenn School of Medicine; USC (Core PI); USC; USC; Indiana University; Indiana University; UC Irvine; Indiana University; Indiana University; Indiana University; Indiana University; UC San Francisco; UC San Diego; Prevent Alzheimer's Disease 2020; UC San Diego; National Institute on Aging; UC San Francisco; Brown University; National Institute of Mental Health; Cornell University; Johns Hopkins University; Richard Frank Consulting; Prevent Alzheimer's Disease 2020; National Institute on Aging; Oregon Health & Science University; University of Southern California; University of California - San Diego; University of Michigan; Mayo Clinic, Rochester; Baylor College of Medicine; Columbia University Medical Center; Washington University, St. Louis; University of Alabama - Birmingham; Mount Sinai School of Medicine; Rush University Medical Center; Wien Center; Johns Hopkins University; New York University; Duke University Medical Center; University of Pennsylvania; University of Kentucky; University of Pittsburgh; University of Rochester Medical Center; University of California, Irvine; University of Texas Southwestern Medical School; Emory University; University of Kansas, Medical Center; University of California, Los Angeles; Mayo Clinic, Jacksonville; Indiana University; Yale University School of Medicine; McGill Univ., Montreal-Jewish General Hospital; Sunnybrook Health Sciences, Ontario; U.B.C. Clinic for AD & Related Disorders; Cognitive Neurology - St. Joseph's, Ontario; Cleveland Clinic Lou Ruvo Center for Brain Health; Northwestern University; Premiere Research Inst (Palm Beach Neurology); Georgetown University Medical Center; Brigham and Women's Hospital; Stanford University; Banner Sun Health Research Institute; Boston University; Howard University; Case Western Reserve University; University of California, Davis - Sacramento; Neurological Care of CNY; Parkwood Hospital; University of Wisconsin; University of California, Irvine - BIC; Banner Alzheimer's Institute; Dent Neurologic Institute; Ohio State University; Albany Medical College; Hartford Hospital, Olin Neuropsychiatry Research Center; Dartmouth-Hitchcock Medical Center; Wake Forest University Health Sciences; Rhode Island Hospital; Butler Hospital; UC San Francisco; Medical University South Carolina; St. Joseph's Health Care; Nathan Kline Institute; University of Iowa College of Medicine; Cornell University; University of South Florida: USF Health Byrd Alzheimer's Institute; University of California, San Francisco; University of Southern California; UC San Francisco; University of Southern California; Mayo Clinic, Rochester; Brigham and Women's Hospital/ Harvard Medical School; UC Davis; Mayo Clinic, Rochester; UC Berkeley; Washington University St. Louis; Indiana University; Perelman School of Medicine, UPenn; USC; Perelman School of Medicine, University of Pennsylvania; UC San Francisco; Rehabilitation Institute of Chicago, Feinberg School of Medicine, Northwestern University; BWH/HMS (Chair); University of Washington (Chair); Core PI; Mayo Clinic, Rochester (Core PI); University of Southern California; UC San Diego; UC San Diego; UC San Diego; UC San Diego; UC San Diego; UC San Diego; UC San Diego; UC San Francisco; UC San Francisco; UC San Francisco; UC Davis (Core PI); UC San Diego; Mayo Clinic, Rochester (Core PI); Mayo Clinic, Rochester; Mayo Clinic; Mayo Clinic; Mayo Clinic; Mayo Clinic; Mayo Clinic; UC Berkeley (Core PI); University of Michigan; University of Utah; Banner Alzheimer's Institute; Banner Alzheimer's Institute; UC Berkeley; Washington University St. Louis; Washington University St. Louis; Washington University St. Louis; Perelman School of Medicine, UPenn; Perelman School of Medicine, UPenn; Perelman School of Medicine, UPenn; Perelman School of Medicine, UPenn; Perelman School of Medicine, UPenn; USC (Core PI); USC; USC; Indiana University; Indiana University; UC Irvine; Indiana University; Indiana University; Indiana University; Indiana University; UC San Francisco; Department of Defense (retired); University of Southern California; University of California, San Diego; Columbia University Medical Center; Rush University Medical Center; Wien Center; Duke University Medical Center; University of Rochester Medical Center; University of California, Irvine; Medical University South Carolina; Premiere Research Inst (Palm Beach Neurology); University of California, San Francisco; Georgetown University Medical Center; Brigham and Women's Hospital; Banner Sun Health Research Institute; Howard University; University of Wisconsin; University of Washington; Stanford University; Cornell University; Department of Psychiatry, Harvard Medical School, Massachusetts General Hospital, Boston, MA, United States

**Keywords:** mild cognitive impairment, non-invasive brain stimulation, stimulation site, meta-analysis, resting state functional connectivity

## Abstract

**Background:**

Non-invasive brain stimulation (NIBS) has been widely used to treat mild cognitive impairment (MCI). However, there exists no consensus on the best stimulation sites.

**Objective:**

To explore potential stimulation locations for NIBS treatment in patients with MCI, combining meta- and resting state functional connectivity (rsFC) analyses.

**Methods:**

The meta-analysis was conducted to identify brain regions associated with MCI. Regions of interest (ROIs) were extracted based on this meta-analysis. The rsFC analysis was applied to 45 MCI patients to determine brain surface regions that are functionally connected with the above ROIs.

**Results:**

We found that the dorsolateral prefrontal cortex (DLPFC) and inferior frontal gyrus (IFG) were the overlapping brain regions between our results and those of previous studies. In addition, we recommend that the temporoparietal junction (including the angular gyrus), which was found in both the meta- and rsFC analysis, should be considered in NIBS treatment of MCI. Furthermore, the bilateral orbital prefrontal gyrus, inferior temporal gyrus, medial superior frontal gyrus, and right inferior occipital gyrus may be potential brain stimulation sites for NIBS treatment of MCI.

**Conclusion:**

Our results provide several potential sites for NIBS, such as the DLFPC and IFG, and may shed light on the locations of NIBS sites in the treatment of patients with MCI.

## Introduction

Mild cognitive impairment (MCI) is defined as subjective memory impairment without dementia or loss of function (Bruscoli and Lovestone, 2004). Literature suggests that MCI can be the early expression of Alzheimer’s disease (AD) and that up to 14.9% of MCI patients older than 65 will develop dementia. However, pharmacologic treatment for MCI is far from satisfactory (Petersen et al., 2018).

Recently, non-invasive brain stimulation (NIBS), such as repetitive transcranial magnetic stimulation (rTMS) and transcranial direct/alternating current stimulation (tDCS/tACS), has been widely used in the treatment of MCI (Brunoni et al., 2014; Nardone et al., 2014; Birba et al., 2017; Padala et al., 2018; Rajji, 2019). While most NIBS studies have targeted brain areas such as the dorsolateral prefrontal cortex (DLPFC), inferior frontal gyrus (IFG), inferior parietal lobule (IPL), and superior temporal gyrus (STG) (Elder and Taylor, 2014; Cappon et al., 2016; Birba et al., 2017), no stimulation location has been universally agreed upon (Pievani et al., 2017).

With the aid of cutting edge brain-imaging tools, investigators have found that many brain regions and networks are involved in the pathology of MCI (Cai et al., 2017; Melrose et al., 2018). These findings provide a basis for exploring new stimulation locations for NIBS. However, some of these regions pose a challenge for NIBS application, as they are located beneath the brain surface and are therefore difficult to access (Pievani et al., 2017).

In past decades, resting state functional connectivity (rsFC) has been widely used in brain research and in identifying NIBS locations. Functional connectivity is defined as the temporal correlation or coherence of a neurophysiological index measured in different brain areas that may show similar functional properties among these brain regions (Friston et al., 1993; Biswal et al., 1997). In a previous study, Fox used a connectivity-based approach to explore the underlying mechanisms of different TMS targets, which demonstrated the potential of using a connectivity-based targeting strategy to optimize TMS locations and increase clinical response (Fox et al., 2012).

In this study, we combined brain imaging meta- and rsFC analyses to explore brain locations for NIBS treatment of MCI. Specifically, we first performed a meta-analysis to identify surface brain areas associated with MCI and then selected these regions as potential NIBS targets. Further, we selected the key MCI-associated brain areas (including both superficial and deep structures) as regions of interest (ROIs) based on the meta-analysis. These ROIs were then used as seeds in the following rsFC analysis to further identify the functionally connected brain regions that are proximal to brain surface cortex in a cohort of MCI patients. We hypothesized that areas on the brain surface that are functionally connected with the deep brain structures involved in MCI pathology (meta-analysis map) may also be used as NIBS target locations in MCI treatment.

## Materials and Methods

In the present study, we first used Neurosynth^1^ to conduct a forward inference meta-analysis at the threshold of *p* < 0.01 FDR-corrected and identify key brain regions involved in the pathophysiology of MCI. Neurosynth is a platform for automatically synthesizing the results of many different neuroimaging studies. Using the Neurosynth framework, a researcher can conduct large-scale automated neuroimaging meta-analyses of broad psychological concepts. The ability of Neurosynth to quantitatively distinguish forward inference from reverse inference allows researchers to assess the specificity of mappings between neural and cognitive functions (Yarkoni et al., 2011).

We then used three different pipelines (methods) to explore potential locations for NIBS in MCI patients. In Pipeline 1, we directly selected clusters on the brain surface of the original forward inference map from the meta-analysis as potential NIBS target regions. In Pipelines 2 and 3, we applied seed-based rsFC analysis to further explore the potential brain surface locations for NIBS in MCI patients. To do this, we first chose 20 ROIs from the meta-analysis map. In Pipeline 2, the group-level correlation maps of each ROI were saved to a binary mask (with positive and negative functional connectivity separated). The 20 positive and 20 negative correlation maps were added to obtain third level maps, and 4–6 surface clusters were selected from the third level maps as potential stimulation locations. In Pipeline 3, after 20 ROIs were picked, we combined them into a single ROI. The group-level seed-based functional connectivity map was created, and 4–6 surface clusters were chosen as potential stimulation locations.

### Meta-Analysis (Pipeline 1)

In the Neurosynth platform, we used “MCI” as the search term to conduct forward inference analysis on all neuroimaging studies in the database that were available until February 2018. The forward brain maps were downloaded from the platform. Potential NIBS locations were chosen based on the report, and they were visually confirmed using BrainNet view^2^ (Xia et al., 2013), as well as MRcon_GL and MRcron_ice toolboxes. These potential locations were mapped onto a standard brain map and standard head map with the international 10–20 system in MNI space (Cutini et al., 2011).

### Resting State Functional Connectivity Analysis

#### fMRI Data Acquisition

The structural and resting state functional MRI data of MCI patients used in the present study were obtained from large multicenter Alzheimer’s Disease Neuroimaging Initiative (ADNI) studies^3^. The ADNI was launched in 2003 as a public-private partnership, led by Principal Investigator Michael W. Weiner, MD. The primary goal of ADNI has been to test whether serial magnetic resonance imaging (MRI), positron emission tomography (PET), other biological markers, and clinical and neuropsychological assessments can be combined to measure the progression of MCI and early AD. A total of 45 MCI patients were included.

The inclusion and exclusion criteria for MCI patients are available at: http://adni.loni.usc.edu/methods/documents/ (ADNI 1 Procedures Manual). Inclusion criteria was as follows: memory complaint by subject or study partner that is verified by a study partner; abnormal memory function documented by scoring below the education-adjusted cutoff on the logical memory II subscale (delayed paragraph recall) from the Wechsler memory scale-revised (the maximum score is 25): (a) less than or equal to 8 for 16 or more years of education, (b) less than or equal to 4 for 8–15 years of education, (c) less than or equal to 2 for 0–7 years of education; mini-mental state exam score between 24 and 30 (inclusive) (Exceptions may be made for subjects with less than 8 years of education at the discretion of the project director); clinical dementia rating = 0.5 Memory Box score must be at least 0.5; General cognition and functional performance sufficiently preserved such that a diagnosis of AD cannot be made by the site physician at the time of the screening visit.”

The data of Mild cognitive impairment patients from 55 to 90 years old with the same fMRI scanning parameters (TR = 3000 ms, TE = 30 ms, phases = 140 with eyes opened) was used in the present study. Eligibility criteria included MCI at any stage.

#### Resting State Functional Connectivity Analysis

Seed-to-voxel correlational analysis were calculated in MATLAB by applying the functional connectivity (CONN) toolbox v17.C^4^. Similar to our previous studies (Tao et al., 2016, 2019; Liu et al., 2019a, b), the images were preprocessed with slice timing, realigned, co-registered to subjects’ respective structural images, normalized, and smoothed with a 6 mm full width at half maximum kernel. Segmentation of gray matter, white matter, and cerebrospinal fluid for the removal of temporal confounding factors was employed. Band-pass filtering was performed with a frequency window of 0.01 to 0.089 Hz.

To eliminate correlations caused by head motion and artifacts, we identified outlier time points in the motion parameters and global signal intensity using ART^5^. We treated images as outliers if the composite movement from a preceding image exceeded 0.5 mm or if the global mean intensity was greater than three standard deviations from the mean image intensity. In addition, the 12 motion regressions (three rotation and three translation parameters plus six first-order temporal derivatives) were used in the preprocessing of BOLD time courses. Outliers were included as regressors in the first level general linear model along with motion parameters (Tao et al., 2016; Wang et al., 2017).

Based on the results of the meta-analysis, we chose the key peak MNI coordinates from the brain map (see details in section “Results” and Table 2) as ROIs. To ensure only voxels that were part of the original Neurosynth map were included, and to maintain regional specificity within the mentioned peak coordinates, the mask was derived by taking the overlap of the original forward inference Neurosynth map and a 6 mm radius spherical mask centered on the identified peak coordinates. This methodology is similar to that of a previous study conducted by Goldstein-Piekarski et al. (2018). The ROIs were further defined and refined by WFU-Pick Atlas software (Maldjian et al., 2003, 2004) to ensure that the regions were within the specific structure identified. The BOLD time course from these ROIs was then extracted, and Pearson’s correlation coefficients were computed between that time course and the time courses of all other voxels in the brain. Correlation coefficients were Fisher transformed into *Z* scores.

Before the rsFC group analysis, we created a brain cortex mask to exclude sub-cortex brain regions from the AAL template using WFU-Pick Atlas software. The mask included bilateral pre and postcentral, superior and middle frontal, superior and inferior and middle occipital, superior and inferior parietal, supramarginal, angular, superior temporal, superior temporal pole, middle temporal, middle temporal pole, inferior temporal, oper inferior frontal, oper Rolandic, tri inferior frontal, superior medial frontal, calcarine, orbital middle and superior and inferior frontal, orbital medial frontal, supplementary motor area, paracentral lobule, precuneus, and cuneus.

##### Pipeline 2

For every seed in the subject-level correlation maps, the residual BOLD time course was extracted for each subject, and Pearson’s correlation coefficients were computed between ROIs and all other brain voxels. The resulting correlation coefficients were subsequently transformed into *Z* scores to increase normality and thus conform to the assumptions of generalized linear models. In the group-level seed-to-voxel analysis, all subject-level seed maps of seed-to-voxel connectivity were included in a one sample *t*-test to obtain a group-level correlation map (positive and negative separated; *Z* values greater than or less than zero). A threshold of voxel-wise *p* < 0.001 uncorrected and cluster-level *p* < 0.05 family-wise error (FWE) corrected were applied within the cortex mask in data analysis.

After all whole-brain rsFC results of all ROIs above threshold had been extracted (binary mask), we calculated the sum of all brain masks using DPABI^6^ to form a correlation map. The intensity of this correlation map represented the number of ROIs, and a higher intensity indicated a greater number of overlapping ROIs in the brain region. The 4 to 6 clusters on the brain surface with the largest peak intensity based on the report, visually confirmed using BrainNet view (Xia et al., 2013) and MRconGL and MRcron_ice toolboxes, were identified as potential stimulation locations.

##### Pipeline 3

We also calculated group functional connectivity using the seed that combined all above seeds into one seed. A threshold of voxel-wise *p* < 0.001 uncorrected and cluster-level *p* < 0.05 FWE corrected were applied within the cortex mask in data analysis.

## Results

### Patient Demographics and Characteristics

Demographic and clinical characteristics of the study group are summarized in Table 1. Of the 45 enrolled MCI patients (24 female), the score of mini mental state examination (MMSE), clinical dementia rating-sum of boxes, and Alzheimer’s disease assessment scale-cognition 12 (ADAS-Cog 12) were 27.64 (1.88), 0.95 (0.78), and 8.68 (3.66), respectively [mean (SD)], with 33.33% APOE4 positive. The average age of MCI patients was 69.32 with a standard deviation of 8.07.

**TABLE 1:** Baseline characteristics.

**Characteristics**	**Mean (SD) *n* = 45**
Age	69.32 (8.07)
Gender (female/male)	24/21
Education (years)	15.71 (2.71)
MMSE	27.64 (1.88)
APOE4+	33.33%
CDR-SOB	0.95 (0.78)
ADAS-Cog12	8.68 (3.66)

*MMSE, mini mental state examination; CDR-SOB, clinical dementia rating – sum of boxes; ADAS-Cog12, Alzheimer’s disease assessment scale-cognition 12.*

### Meta-Analysis Results (Pipeline 1)

**FIGURE 1:**
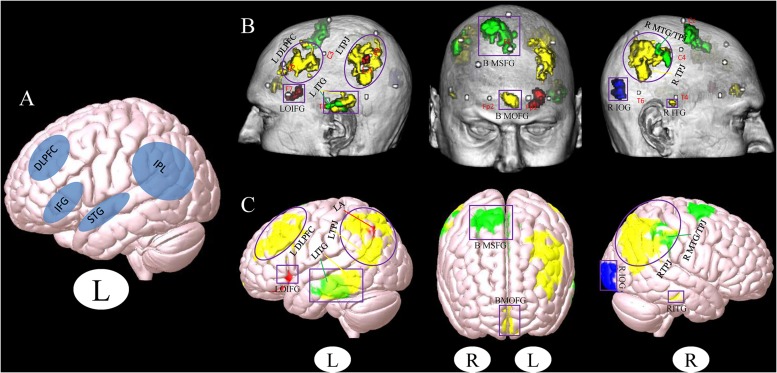
**(A)** Stimulation location setup from a previous review study (Birba et al., 2017). **(B**,**C)** Cluster associated with the brain surface based on the results of meta- and functional connectivity analyses **(B)**, the template of chris_t1.nii.gz was used from Mricro_GL software. **(C)** The template of mni152.2009.mz 3 was used from Surf_Ice software. Red, results from the meta-analysis; Yellow, results from the positive rsFC; Green, results from the negative rsFC; Blue, results from the combination of all ROIs rsFC; Gray, MNI coordinates of 10–20 scalp positions found by Cutini et al. (2011). Purple box indicates the different brain regions between the results of a previous study (Birba et al., 2017) and our present results. Purple circle indicates the overlapping brain regions between the results of a previous study (Birba et al., 2017) and our present results (L, left and R, right). LDLPFC, left dorsolateral prefrontal cortex; LTPJ, left temporoparietal junction; LOIFG, left orbital inferior frontal gyrus; LITG, left inferior temporal gyrus; BMOFG, bilateral medial orbital frontal gyrus; BMSFG, bilateral medial superior frontal gyrus; RMTG/TPJ, right middle temporal gyrus and right temporoparietal junction; RITG, right inferior temporal gyrus; RIOG, right inferior occipital gyrus; LA: left angular.

A total of 67 studies were included in the meta-analysis, and 20 clusters had a size greater than 30 continuous voxels. With the BrainNet viewing software, we visually determined that the left orbital IFG (F7) and left angular gyrus (P3) are proximal to the brain surface and can thereby serve as brain stimulation sites directly (Figures 1B,C, Table 2, and Supplementary Figure S1A).

**TABLE 2:** Results of meta-analysis (^∗^indicates cluster on the brain surface).

**Cluster**	** Voxel**	**Peak intensity**	**Peak MNI coordinate**			**Brain region**
				
			***x***	***y***	***z***	
1	519	6.88	−22	−22	−16	L hippocampus
2	442	10.84	24	−10	−20	R hippocampus
3	80	5.90	−54	−6	−20	L middle temporal gyrus
4	94	6.88	40	−54	−20	R fusiform gyrus
5^∗^	101	6.88	−36	26	−8	L orbital inferior frontal gyrus
6	85	5.90	34	26	−6	R orbital inferior frontal gyrus
7	37	4.91	−12	62	−12	L superior orbital frontal gyrus
8	48	4.91	−6	−58	2	L lingual gyrus
9	39	4.91	6	−18	8	R thalamus
10	436	8.86	0	−54	28	L post cingulum cortex
11	31	7.91	44	34	16	R tri inferior frontal gyrus
12	101	5.90	−50	12	26	L oper inferior frontal gyrus
13	59	5.90	0	54	30	L medial superior frontal gyrus
14	31	4.91	−34	−78	40	L inferior parietal gyrus
15	77	6.88	−42	−46	40	L inferior parietal gyrus
16^∗^	41	5.90	−44	−64	40	L angular gyrus
17	59	6.88	46	−4	44	R precentral gyrus
18	47	5.90	−14	−68	50	L superior parietal gyrus
19	54	5.90	34	−58	50	R angular gyrus
20	43	6.88	10	14	50	R supplementary motor area

*L: left; R: right.*

### Seed-to-Voxel rsFC Results

To further investigate potential surface locations of brain stimulation, we selected the peak coordinates from the meta-analysis maps as ROIs (seeds) (Supplementary Figure S2) and performed a rsFC analysis. The 20 seeds of MNI coordinates (*X*, *Y*, and *Z*) are shown in Table 2.

We used the above 20 seeds and applied rsFC analysis separately. The brain surface regions that showed overlap positively correlated with seed regions are listed in Figures 1B,C, Table 3, and Supplementary Figure S1B. These regions include the left inferior temporal gyrus, right inferior temporal gyrus, bilateral medial orbital frontal gyrus, right middle temporal gyrus/temporoparietal junction (TPJ), left middle occipital gyrus/TPJ, and left DLPFC. The left inferior temporal gyrus (T3), right supramarginal gyrus (between C4 and P4), and bilateral medial superior frontal gyrus (T3 and T4) were found to be negatively associated with seed regions applied in this study (Figures 1B,C, Table 3, and Supplementary Figure S1C; Pipeline 2).

**TABLE 3:** Resting state functional connectivity results of regions on the brain surface.

**Seed**	**Brain region**	**Cluster**	**Peak intensity**	**Peak MNI coordinate**			**Positive rsFC**	**Negative rsFC**
				***X***	***Y***	***Z***		
20 seeds from meta-analysis	L inferior temporal gyrus	526	6	−54	−48	−18	✓	
	R inferior temporal gyrus	34	5	58	−22	20	✓	
	Bilateral medial orbital frontal gyrus	216	6	−2	58	−8	✓	
	R middle temporal gyrus/TPJ	2023	8	54	−60	14	✓	
	L middle occipital gyrus/TPJ	1879	8	−32	−70	28	✓	
	L DLPFC	1093	9	−42	4	38	✓	
	L inferior temporal gyrus	315	4	−52	−28	−18		✓
	R supramarginal gyrus	340	4	58	−28	32		✓
	Bilateral medial superior frontal gyrus	1066	4	−2	18	42		✓
Combinate seeds	R inferior occipital gyrus	277	4	38	−90	−14	✓	

*L: left; R: right; TPJ: temporoparietal junction; DLPFC: dorsolateral prefrontal cortex.*

When combining all seeds into one large seed, we only found positive rsFC with the right inferior occipital gyrus (between T6 and O2) proximal to the brain surface (Figures 1B,C, Table 3, and Supplementary Figure S1D; Pipeline 3).

## Discussion

In this study, we combined a meta-analysis and rsFC analysis to explore potential stimulation locations at the brain surface in patients with MCI. Our results suggest that the left DLPFC, left inferior orbital frontal gyrus, bilateral inferior temporal gyrus, TPJ, medial superior frontal gyrus, medial orbital prefrontal gyrus, right middle temporal gyrus, and inferior occipital gyrus should be considered as potential brain stimulation sites for MCI.

Location is a critical component in the treatment of MCI via NIBS (Fox and Greicius, 2010; Bergmann et al., 2016). In a previous study, Birba et al. (2017) used a meta-analysis method to test the effects of NIBS techniques on patients with MCI. They found that the DLPFC and IFG are brain stimulation areas commonly used in studies (Figure 1A). These findings are consistent with our results, indicating that these regions should be preferentially selected in the treatment of MCI with NIBS.

In further support of the important role of the DLPFC in patients with MCI, Yang et al. (2009) found that the BOLD response of the DLPFC is weaker in patients with MCI when compared with normally aging individuals. This finding suggests that a functional abnormality of the DLPFC may be an early indicator of the disease. In addition, results from neuroimaging studies suggest that the IFG plays an important role in cognitive functions such as memory processing (Thompson-Schill et al., 1997). For instance, Bell reported that, compared with amnestic MCI patients, patients with MCI-multiple cognitive domain had significantly reduced volume of the right IFG (Bell-McGinty et al., 2005). Lin found that IFG activities may protect the memory function of individuals with MCI (Lin et al., 2017). These studies demonstrate the functional alterations of the DLPFC and inferior prefrontal gyrus in MCI and provide support for using these regions as treatment targets.

The temporal gyrus/TPJ was an overlapping brain region of both the meta-analysis and rsFC analysis, suggesting that it may be another important stimulation site for patients with MCI. A previous study reported that, compared with healthy elderly subjects, amnesiac MCI (aMCI) patients showed increased ALFF values in the left TPJ (Xi et al., 2013). The TPJ has been implicated across a broad range of cognitive areas, such as attention, social cognition, decision making, and episodic memory reconsolidation (Simon et al., 2017). Olichney reported that there was a significant suppression in amplitude of the late positive potential in the TPJ during a 3-choice vigilance task in individuals with MCI, and this change in amplitude may be a potential biomarker for early dementia (Olichney et al., 2011). Further, a study from Griffith et al. (2010) reported that the volume of the angular gyrus, a brain area within the TPJ, can predict cognitive function in MCI patients. Herholz also found that non-pharmacological interventions like recreational activity can yield an increase in cortical thickness of the right angular gyrus in patients with early stage AD (Herholz et al., 2013).

Our results suggest that the bilateral orbital prefrontal gyrus, inferior temporal gyrus, medial superior frontal gyrus, and right inferior occipital gyrus are potential stimulation sites based on rsFC analysis. Previous studies have suggested that the orbitofrontal cortex is important in cognitive processes such as learning, decision making (Wilson et al., 2014), and maintaining appropriate social behavior (Jonker et al., 2015). Sacuiu showed that cortical atrophy in the frontal lobe, including the orbital prefrontal gyrus, can hasten the conversion of MCI to AD (Sacuiu et al., 2016).

In addition, studies suggest that the inferior temporal gyrus plays an important role in verbal fluency processing (Scheff et al., 2011) and serial visual recognition (Baylis and Rolls, 1987). For instance, investigators found that synaptic loss in the inferior temporal gyrus is a prominent pathological defect in the early stages of AD, and individuals with aMCI have significantly fewer synapses compared to those with no cognitive impairment (Scheff et al., 2011). The current literature suggests that increased activity of the superior frontal gyrus may be closely related to attentional shift between object features (Nagahama et al., 1999), cognitive control, working memory, and execution (Zhang et al., 2012).

Moreover, the medial superior frontal is an important node within the default mode network (DMN). Zhou et al. (2015) found several important nodes within the DMN that are involved in MCI, including the medial superior frontal gyrus. Huang et al. (2016) suggested that the DMN may indeed be a crucial target for interventions of MCI. Fink et al. (1996) found that the left inferior occipital cortex was activated in a local directed attention task, and Belleville suggested that attentional control deficits occur in patients with MCI (Belleville et al., 2007). Taken together, these studies provide further support for the aforementioned brain regions as targets for MCI treatment with NIBS.

It is worth noting that identifying these locations may also help to advance other interventions such as scalp acupuncture. Unlike traditional acupuncture, where needles are inserted into acupuncture points, scalp acupuncture stimulates the area of scalp corresponding to brain regions believed to be involved in the pathology of the disorders (Hao and Hao, 2012). Thus, results obtained from this study may facilitate the development of acupuncture and therapeutic modalities such as transcutaneous electrical nerve stimulation for the treatment of MCI.

Further, the aim of this preliminary study was to investigate potential stimulation locations for NIBS treatment using a unique approach that combined meta- and functional connectivity analyses. However, different stimulation techniques may have different intensities and reach different depth. How to apply and optimize these different treatment modalities to target the brain areas identified in our study is beyond the scope of this manuscript. Investigators should consider the characteristics of different tools when attempting to stimulate these areas.

## Conclusion

Our results suggest that the DLPFC, IFG, and TPJ should be preferentially recommended as locations for NIBS treatment of MCI. Furthermore, the bilateral orbital prefrontal gyrus, inferior temporal gyrus, medial superior frontal gyrus, and right inferior occipital gyrus may also be potential brain stimulation sites. Our results ultimately shed light on possible NIBS sites in the treatment of MCI and other cases of age-related cognitive decline.

## Limitations

There are several limitations to our study. First, while we provide several potential stimulation sites for NIBS treatment, we cannot predict the specific effects of such stimulation. For instance, we do not know whether the effect of the stimulation will be excitatory or inhibitory in nature. Another limitation is that the results of the connectivity analysis were strictly functional in the present study (i.e., rsFC). Future studies combining anatomical and functional connectivity analyses may yield stronger evidence for stimulation sites. Furthermore, our present study provided only a starting point for brain stimulation location selection via meta- and rsFC analysis methods. Determining the best brain region for NIBS remains a challenge. One option is to apply stimulation to multiple regions simultaneously. Stimulation paradigms are beyond the scope of this manuscript, and future studies are needed to explore these paradigms based on the specific NIBS modalities and, most importantly, the condition of the individual. In addition, due to the database limitation, we included patients within the age range of 55–90. Future studies with a smaller age range should be conducted to reduce the effects of standard deviation of age.

## Data Availability

All datasets generated for this study are included in the manuscript and/or the Supplementary Files.

## Ethics Statement

Data used in preparation of this article were obtained from the Alzheimer’s disease ADNI database (adni.loni.usc.edu). As such, the investigators within the ADNI contributed to the design and implementation of ADNI and/or provided data but did not participate in analysis or writing of this report. A complete listing of ADNI investigators can be found at: http://adni.loni.usc.edu/wp-content/uploads/how_to_apply/ADNI_Acknowledgement_List.pdf.

## Author Contributions

JK designed the experiments. JL, BZ, and JK analyzed and interpreted the data. JL and GW prepared the manuscript. All authors prepared the manuscript, and read and approved the final manuscript.

## Conflict of Interest Statement

JK has a disclosure to report (holding equity in a startup company, MNT, and pending patents to develop a new brain stimulation device) but declares no conflict of interest. The remaining authors declare that the research was conducted in the absence of any commercial or financial relationships that could be construed as a potential conflict of interest.

## Footnotes

1 http://neurosynth.org/

2 https://www.nitrc.org/projects/bnv/

3 http://adni.loni.usc.edu/

4 http://www.nitrc.org/projects/conn

5 http://www.nitrc.org/projects/artifact_detect

6 http://rfmri.org/dpabi

## Supplementary Material

FIGURE S1 **(A)** Results from the meta-analysis. **(B)** Results from the positive rsFC based on Pipeline 2 analysis. **(C)** Results from the negative rsFC based on Pipeline 2 analysis. **(D)** Results from the combination of all ROIs based on Pipeline 3 analysis.

FIGURE S2 ROIs used for the functional connectivity analysis.
