# Supplementary figures and images for: New Perspective for Non-invasive Brain Stimulation Site Selection in Mild Cognitive Impairment: Based on Meta- and Functional Connectivity Analyses
Source: Front Aging Neurosci. 2019 Aug 27;11:228. doi: 10.3389/fnagi.2019.00228 (PMC6736566; doi:10.3389/fnagi.2019.00228)

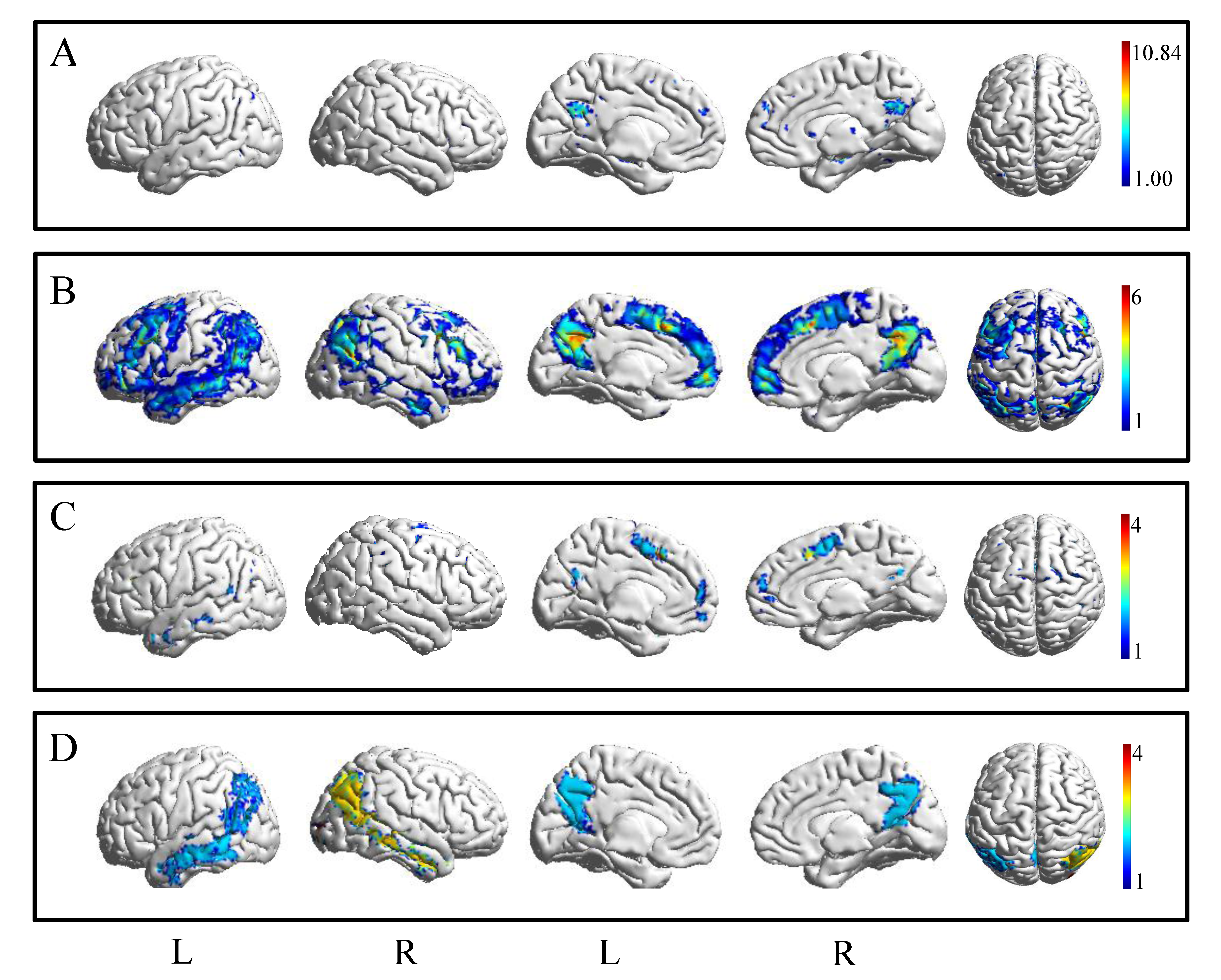

Supplement: FIGURE S1 — (A) Results from the meta-analysis. (B) Results from the positive rsFC based on Pipeline 2 analysis. (C) Results from the negative rsFC based on Pipeline 2 analysis. (D) Results from the combination of all ROIs based on Pipeline 3 analysis. [file Image_1.TIF]

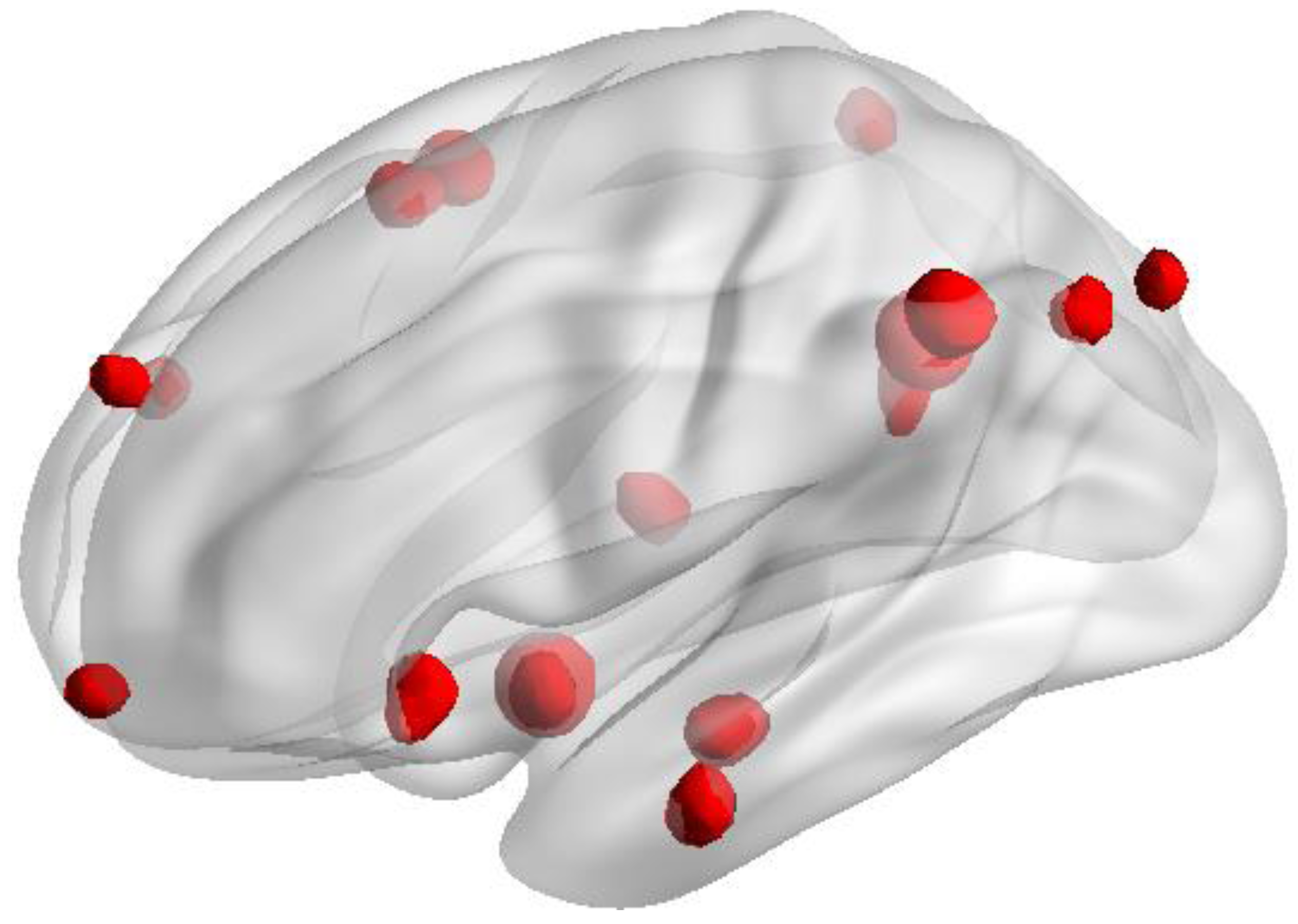

Supplement: FIGURE S2 — ROIs used for the functional connectivity analysis. [file Image_2.TIF]
